# A retrospective study on acute health effects due to volcanic ash exposure during the eruption of Mount Etna (Sicily) in 2002

**DOI:** 10.1186/2049-6958-8-51

**Published:** 2013-08-07

**Authors:** Daniele Lombardo, Nicola Ciancio, Raffaele Campisi, Annalisa Di Maria, Laura Bivona, Venerino Poletti, Antonio Mistretta, Annibale Biggeri, Giuseppe Di Maria

**Affiliations:** 1 Department of Clinical and Molecular Biomedicine, University of Catania, Catania, Italy; 2 Pulmonology Unit, A.O.U. Policlinico-Vittorio Emanuele, Catania, Italy; 3 Department of Diseases of the Thorax, Pulmonology Unit, Pierantoni-Morgagni Hospital, Forlì, Italy; 4 Department of Hygiene and Public Health “G.F. Ingrassia”, University of Catania, Catania, Italy; 5 Department of Statistics, Informatics and Applications “G. Parenti”, University of Florence, Florence, Italy; 6 Biostatistics Unit ISPO Cancer Prevention and Research Institute, Florence, Italy

**Keywords:** Acute health effects, Ash fall, Cardiovascular effects, Emergency department visits, Respiratory effects, Volcanic eruption

## Abstract

**Background:**

Mount Etna, located in the eastern part of Sicily (Italy), is the highest and most active volcano in Europe. During the sustained eruption that occurred in October-November 2002 huge amounts of volcanic ash fell on a densely populated area south-east of Mount Etna in Catania province. The volcanic ash fall caused extensive damage to infrastructure utilities and distress in the exposed population. This retrospective study evaluates whether or not there was an association between ash fall and acute health effects in exposed local communities.

**Methods:**

We collected the number and type of visits to the emergency department (ED) for diseases that could be related to volcanic ash exposure in public hospitals of the Province of Catania between October 20 and November 7, 2002. We compared the magnitude of differences in ED visits between the ash exposure period in 2002 and the same period of the previous year 2001.

**Results:**

We observed a significant increase of ED visits for acute respiratory and cardiovascular diseases, and ocular disturbances during the ash exposure time period.

**Conclusions:**

There was a positive association between exposure to volcanic ash from the 2002 eruption of Mount Etna and acute health effects in the Catania residents. This study documents the need for public health preparedness and response initiatives to protect nearby populations from exposure to ash fall from future eruptions of Mount Etna.

## Background

There are a number of active volcanoes throughout the world and many of them are close to urban settings and major cities. At times, they are responsible for pyroclastic emissions, consisting of a mixture of gases, vapours, aerosols, and particulate matter. These emissions have the potential to cause massive environmental pollution and impact on climate, surface infrastructures and human activities, thus resulting in a significant economic burden for both the community and the individuals [1]. Among the adverse effects of pyroclastic emissions (ash fall), those related to human health raise significant concern in both exposed populations and health service administrators [1,2].

Previous observations report that although volcanic ash may not be acutely toxic, its exposure has been associated with a variety of health effects - spanning from sudden, asphyxia-induced death due to acute respiratory tract irritation and symptoms either in subjects with existing respiratory disorders like asthma or healthy individuals [3-6]. Adverse respiratory effects from exposure to volcanic ash have been documented during the eruption of Montserrat, British West Indies, in 1995 [7,8], and from Eyjafjallajökull volcano, Iceland, in 2010 [9,10]. In addition, repeated exposure to ash fall from Mount Sakurajima, Japan, was associated with increased mortality due to respiratory diseases including lung cancer and chronic obstructive pulmonary disease (COPD) [11].

Despite the fact that volcanic eruptions are common throughout the world, and millions of people are exposed to their health hazards, few studies have been published that assess acute and chronic health effects thereby shaping prevention and measures of intervention for local communities.

**Figure 1:**
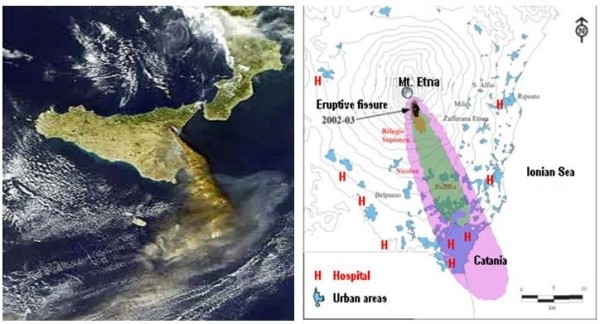
**Area interested by the eruption and ashfall from satellite, with indication of the Hospital (H) reported in the study.** Local winds carried the emissions for kilometers and affected a largely populated district extending south of Mount Etna.

Mount Etna, located in the eastern part of Sicily (Italy), is the most active volcano in Europe. In 2002 an intense eruption activity began in October and ended up November 2002 [12]. This long lasting eruption produced explosions and jets of lava from two newly formed fissures in the proximity of the volcano’s summit, and it was also characterized by the emission of pyroclastic flows that were more intense during the first two weeks of activity [12]. Local winds carried the emissions for kilometers and subsequent ash fall affected a largely populated area extending south of Mount Etna, which includes Catania, a major city with nearly 315,000 inhabitants (Figure 1). During the eruption the exposed population complained of health disturbances such as eye and airway irritation, cough, or acute exacerbation of chronic respiratory disorders. However, a subsequent study investigating the rate of hospital admissions and causes of death only found an increase in cardiovascular diseases along with a decrease in mortality for respiratory diseases during the eruption period in the same area [13,14].

To ascertain whether or not there was an association between the Mount Etna's ash fall and acute health hazards in the exposed communities, we carried out a retrospective study to investigate the number and type of visits to the emergency departments (ED) of hospitals near Mount Etna during the 2002 eruption.

## Methods

To test the hypothesis that there is a putative association between acute health effects reported by the exposed population and the volcanic ash fall, we collected the number and type of visits to the ED for various acute diseases in public hospitals (see end-note) near the volcano in the Province of Catania between October 20 and November 7, 2002. We compared these results with the number of ED visits during the corresponding period in 2001. Disease conditions were classified according to previous surveillance activity that was conducted during the eruption as required by the Prefecture of Catania.The surveillance activity was aimed to detect and monitor acute health effects in order to provide prompt intervention to exposed residents. These conditions included acute respiratory disorders of both the upper and lower respiratory tract, cardiovascular diseases such as heart attack, coronary and cerebrovascular diseases, arterial hypertension and cardiac arrhythmias, as well as ocular disturbances, neuropsychiatric disorders, skin diseases and traumatic events. The outcome measure was the proportion of ED visits for those conditions among the total ED visits, whereas the effect measure was the ratio between the proportion in the exposed time period during 2002 to the proportion in same yet unexposed time period during 2001. Whereas the population denominators were constant for the two considered periods, the total number of ED visits changed because of a change in policies of the healthcare system in Italy. We therefore used the proportion instead of the rate of ED visits for the selected disease states [15].

In addition to health monitoring, levels of particulate matter ≤10 μm (PM_10_), and 2.5 μm (PM_2.5_) in diameter were also recorded in the same geographic area near Mount Etna [13,14].

The chemical composition of volcanic ash and the amount of each surface component were analyzed by mass spectrometry.

Statistical analysis was performed to quantify and verify the relevance of differences between the two study periods. Statistical tests used were the “Chi-Square (χ^2^)” and the “Z test” with a two-sided α =0.05. We also applied multinomial logistic regression to adjust for hospital of admission on a restricted sample of 73% of ED visits with complete information. This model produced exposure Odds Ratio (OR) estimates. The reference category was the number of ED visits for all other diseases. For example, an exposure OR of 1.30 for acute respiratory disease would be interpreted as an increase of 30% in the odds of having a ED visit for acute respiratory disease versus the odds of having an ED visit for a condition not related to volcanic ash exposure.

## Results

We first summarize the results of chemical analysis of volcanic ash sampled in Catania from this eruption. Whereas, in the second part we report the results of acute health effects during the intense ash fall period.

### Analysis of volcanic ash

The Protezione Civile, the Italian organization for disaster management, estimated that four days after the beginning of eruption, 9 kg of ash per square meter had fallen in Nicolosi (a small town 15 km south of the volcano), and 2.5 kg per square meter in Catania (24 km south-east) [7].

The chemical analysis carried out by mass spectrometry on ash samples of October 28, 2002, indicates that the fine particles of volcanic ash were mainly composed of ferrous ions (Fe^++^) and a great presence of free crystalline silica in large particles.

### Acute health effects

The results of this retrospective study are presented in Table 1, which shows the frequency and level of statistical significance in the proportion of ED visits to the Hospitals in the Province of Catania - for disease states and health effects that may be related to the exposure to volcanic ash emission. The difference in the proportion of selected health effects varied among the different hospitals for each selected disease category. There was a significant increase in the number of ED visit for selected disease states in the three main hospitals serving the municipality of Catania, i.e. Vittorio Emanuele, Cannizzaro and Garibaldi Hospital. These hospitals were located in the area that received the plume of ash fall (Figure 1). Taking into account the data obtained from the EDs of these three main hospitals only, the cumulative number of total visits in 2002 was less than the corresponding number in 2001 (10,337 and 12,230, respectively). Despite this, the proportion of ED visits for the selected categories was significantly higher with the exclusion of neuropsychiatric disorders and skin diseases (Table 2). Therefore, a significant increase in the number of visits for acute diseases of upper and lower respiratory tract, cardiovascular diseases and ocular symptoms was observed between October 20 and November 7 2002 compared to the corresponding period of 2001.

**Table 1:** Level of significance and difference in the frequency, in ED visits of the hospitals in the province of Catania, for some acute diseases

	**Vittorio Emanuele Hospital**		**Cannizzaro Hospital**		**Garibaldi Hospital**		**Hospital of Giarre**		**Hospital of Militello**		**Hospital of Acireale**		**Hospital of Paternò**		**Hospital of Biancavilla**		**Hospital of Bronte**	
	**Differ.**	**Value P**	**Differ.**	**Value P**	**Differ.**	**Value P**	**Differ.**	**Value P**	**Differ.**	**Value P**	**Differ.**	**Value P**	**Differ.**	**Value P**	**Differ.**	**Value P**	**Differ.**	**Value P**
Acute affections of the upper respiratory tract	+2,73	p < 0,01	+0,17	NS	−0,10	NS	+0,2	NS	−0,27	NS	−4,70	NS	−0,15	NS	−4,82	P < 0,05	−0,34	NS
Acute diseases of lower respiratory tract	+1,45	p < 0,01	+1,24	p < 0,01	+0,10	p < 0,01	+3,11	p < 0,01	−0,50	NS	+0,09	NS	−0,32	NS	−0,63	NS	−0,48	NS
Cardiovascular diseases	+1,18	p < 0,01	+2,99	p < 0,01	−0,01	NS	+1,7	P < 0,05	−0,30	NS	+7,28	P < 0,05	+0,56	NS			−0,61	NS
Neuropsychiatric disorders	−0,08	NS	−0,01	NS	+0,17	NS	+1,17	NS			−0,16	NS	−0,13	NS	+0,07	NS	+0,82	NS
Ocular affections	+2,02	p < 0,01	−0,60	P < 0,05	+2,6	p < 0,01	+0,16	NS			−1,83	NS	−0,05	NS			+0,05	NS
Skin diseases	+0,2	NS	−0,08	NS	−0,33	NS	−1,9	p < 0,01	−0,46	NS	−0,472	NS	−0,44	NS	−0,23	NS	−0,15	NS
Surgery and orthopedy	−1,10	NS	+4,36	p < 0,01	+0,06	NS	+5,62	P < 0,05	−0,84	NS	−1,52	NS	+1,98	p < 0,01	−2,55	NS	+0,68	NS

Legend:

p < 0,01 highly significant difference.

p < 0,05 statistically significant difference.

p > 0,05 not significant difference.

The period is between October 20 and November 7, 2002. The results obtained were compared with those of the same period in 2001. Table 2 contains more detailed information in relation to the three major hospitals in Catania.

**Table 2:** Number and frequency of visits, to ER of the Hospitals Vittorio Emanuele, Cannizzaro and Garibaldi in Catania, for some acute affections

**Visits to ER – observation period 20/10 - 05/11**			**Differ**	**Value P**
**City of Catania**	**Year 2001**	**Year 2002**		
N. acute affect.upper resp. tract (% tot.)	262 (2,14)	353 (3.41)	(+1,27)	p <0,01
N. acute affect. lower resp. tract (% tot.)	256 (2,09)	350 (3,38)	(+1,29)	p <0,01
N. heart diseases (% tot.)	182 (1,49)	287 (2,78)	(+1,29)	p <0,01
N. neuropsychiatric disorders (% tot.)	139 (1,14)	116 (1,12 )	(−0,02)	NS
N. ocular affections (% tot.)	292 (2,39)	409 (3,96)	(+1,57)	p <0,01
N. skin diseases (% tot.)	106 (0,86)	86 (0,83)	(−0,03)	NS
N. surgical diseases and orthopedic (% tot.)	1157 (9,46)	1048 (10,14)	(+0,68)	NS
Total diseases	2394 (1,96)	2649 (2,56)	(+0,60)	
Number of visits for all diseases (total)	12.230	10.337		

Legend:

p < 0,01 highly significant difference.

p < 0,05 statistically significant difference.

p > 0,05 not significant difference.

We also analyzed the frequency of visits to ED of the Hospitals in Catania through multinomial logistic regression adjusting for hospital of admission, but we found no difference from previous analyses (Table 3).

**Table 3:** OR trough polytomic multinomial logistic regression

**Visits in ER**	**OR**	**Std. error**	**z**	**P > [z]**	**[95% conf. interval]**
Acute respiratory diseases	1.3046	0.1136	3.05	0.002	1.0998 – 1.5475
Heart diseases	1.3600	0.1128	3.71	0.0001	1.1560 – 1.6001
Ocular affections	1.1885	0.1046	1.96	0.050	1.0002 – 1.4123
Trauma	1.2247	0.0713	3.48	0.001	1.0925 – 1.3728

Number of observations = 16.069.

## Discussion

According to current knowledge volcanic ash creates minor health problems such as eye and upper airway irritation, and may exacerbate pre-existing respiratory diseases including asthma and chronic bronchitis. In this study we found that during the sustained pyroclastic emission of volcanic ash due to Mount Etna eruption in 2002, ED visits for acute health disorders of respiratory tract and cardiovascular system increased significantly. This increase suggests that volcanic ash fall is associated with acute health effects on exposed local communities, and confirms the results of previous studies showing that short-term exposure to volcanic emission is associated with acute respiratory morbidity, asthma attacks, and increased hospital visits for respiratory illness [3-6,9], as well as for cardiovascular symptoms and morbidity [14,16].

Due to the lack of information on time relationship between daily levels of particulate matter and daily rate of ED visits we were unable to perform time-series analysis of our data. Consequently, we cannot conclude that a causal relationship exists between exposure to volcanic ash and health effects observed in our study. The existence of an association, however, is further supported by the evidence that unusually high levels of respirable sized particulate matter, along with a nearly threefold increase in PM_10_ and fourfold increase in sulfur dioxide, were recorded during the same period in the same area as compared to the previous year [13]. Contrary to the results reported by Fano and coworkers [13,14] of an increase in hospital admissions for cardiovascular morbidity along with a decrease in the rate of admission for respiratory diseases, we found a significantly higher frequency of ED visits for both cardiovascular and respiratory diseases in the three main hospitals of Catania in 2002 as compared to the same period of the previous year. By taking into account that hospital admissions represent a higher intensity of care than an ED visit, a reason for this discrepancy could rely upon a precautionary attitude of ED doctors towards the hospital admission of patients with cardiovascular symptoms rather than of those presenting with respiratory symptoms. On the other hand, the inclusion in our selection of minor respiratory problems, such as the upper airway irritation, and the higher number of hospitals considered for the rate of hospitalization might represent further explanation for the different behavior of hospital admissions and ED visits for acute respiratory diseases. In addition, our data do not provide crude figures of mortality, nor they allow for the stratification of age range, gender, smoking habit, occupational risks, and pre-existing disease states.

Volcanic eruptions are relatively frequent throughout the world, yet few other observational studies have been carried out on this topic. In addition, besides the scarcity of published studies, researchers have investigated ash fall and associated health effects by using varied approaches thus making the broad interpretation of findings difficult or not fully comparable. For example, some studies analyzed hospital admissions or ED visits, rather than outpatient or GP visits. In Alaska during the volcanic activity of Mount Spurr in 1992, located 60 miles west of the city of Anchorage, an increase in PM_10_ along with an increase for outpatients visits for bronchial asthma and upper respiratory diseases was observed [17]. Few investigations with conflicting data are available on mortality rate during or after eruptions, as well as on the long-term effects of volcanic ash exposure. No effects on overall and cause-specific mortality were observed during the eruption of Mount Etna in 2002, as reported by a different group of investigators [13,14]. By contrast, residents in Sakurajima-Tarumizu area located near Mt. Sakurajima in Japan, over the long term (>30 years) experienced a relatively high mortality for respiratory diseases, which included lung cancer and COPD [11]. In 6–9 months after the end of Eyjafjallajökull eruption, residents from exposed areas reported several medical problems including increased wheezing, cough and phlegm, as well as eye and skin irritation [10].

Another problem is to determine whether or not some working categories, such as local police, firefighters or people involved in extensive ash-removal operations, have a higher level of exposure and maybe a higher risk for developing acute health effects. During the eruption of Mount St. Helens, on May 18, 1980 respiratory symptoms and ocular problems were significantly higher among loggers who were regularly employed outdoors in areas with heavy ash exposure, than among other groups of workers who weren’t assigned to clean up after volcanic eruptions. Loggers inhaled higher concentration of ash showing a significant, short-lived reduction in lung function. This transitory functional impairment was directly proportional to the intensity of exposure to volcanic ash [2-4]. One reason for the observed higher risk of respiratory illness among population involved in ash-removal operations may be due to the “re-suspension” of fine ash particles and their chemical components. However, pragmatically, this distinction may not make much difference. A proportion of silica particles detected during Mount Etna’s eruption were small enough to penetrate deeply into the lungs of exposed subjects. It is conceivable that re-suspension caused by atmospheric factors or human activity such as road traffic and ash removal may have re-suspended particles with smaller diameter and more likely to be inhaled, thus determining an increase in exposure. Soundly, this is not trivial and suggests the opportunity to adopt strategies avoiding the phenomenon of re-suspension.

Little information is available about the relevance of age, sex, and more importantly the possible presence of pre-existing chronic respiratory diseases in patients that presented to EDs during the 2002 eruptive event at Mount Etna. During the July 1995 eruption in Montserrat, a study was performed with the aim to evaluate whether ash fall had any effect on the respiratory health of children. The results were that volcanic ash emission adversely affected the respiratory health and produced exercise-induced bronchoconstriction in the Montserrat children [7]. The health effects of volcanic ash on the respiratory system might depend on size and composition of the inhaled particles. Their diameter is the most important determinant of penetration into the peripheral airways. The size of 10 μm is considered to be the cut-off point for the aerodynamic diameter of respirable particles. However, penetration to the respiratory bronchioles and alveolar spaces is limited to particles with diameter of 2.5 μm or less.

By reaching the small airways, it is thought that volcanic ash causes airway inflammation, and it has been reported that patients with chronic obstructive pulmonary disease and bronchial asthma, are more prone than healthy people [3].

Referring to traumatic injuries, a further analysis of our data showed that they were significantly reduced in the main Hospital located in the downtown area of Catania. This may be explained by the advice issued by local authorities, that was not extended to neighboring communities, about risks of driving motorcycles on ash-laddened roads in downtown Catania city.

Other adverse effects of volcanic ash include the irritation of upper respiratory tract causing nasopharyngitis, with transient hyperemia, as well as eye irritation causing conjunctivitis. Our data are in agreement with those reported in previous studies [9,10].

Finally, it is important to take into account the composition of volcanic ash, and evaluate the possible tumorigenic effects of some ash components. In our experience, during the eruption of Mount Etna in 2002, analysis of large particles performed with a mass spectrometer showed a relevant proportion of free crystalline silica, which upon long-term exposure to particularly high concentrations, could represent a risk factor for the development of pneumoconiosis [18]. Furthermore, the presence of ferrous ions on the surface of fine particles, was registered with the same method. It has been reported that removable divalent iron (Fe^++^), can trigger inflammatory processes causing the release of free radicals in the lung tissue [6]. An *in vitro* study has shown that biochemical processes induced by exposure to high levels of PM_10_ could produce cytotoxicity and DNA alterations [19]. In summary, these observations are highly suggestive that volcanic ash exposure could represent a hazard for developing lung cancer. The existing data on this issue, however, are not conclusive and further studies are needed.

## Conclusions

In conclusion, in this study we found an association between exposure to volcanic ash fall and acute health effects in the resident population of Catania at Mount Etna. Peculiar evolution of the eruption in 2002 and the volcanic and geographical characteristic of the volcano do not provide an “ideal-model” to study health hazards of ash producing eruptions. However, the information provided by this study is useful to assist the Province of Catania in planning environmental and public health measures that could limit exposure and protect the population. Indeed much work remains to determine the specific short-term and long-term health risks volcanoes pose to humans. In the future, it may be necessary to design and implement longitudinal epidemiological observations in areas at high risk of volcanic emissions, to answer the questions still left open by the present and other investigations.

## Endnotes

Denomination of the Hospital cited in the article. The main hospital in the town of Catania are the first three listed.

1) Azienda Ospedali Vittorio Emanuele, Ferrarotto e S. Bambino,

2) Azienda Ospedaliera Cannizzaro,

3) Azienda Ospedali Garibaldi, S. Luigi-Currò, Ascoli-Tomaselli,

4) Presidio Ospedaliero S. Giovanni di Dio e S. Isidoro di Giarre,

5) Presidio Ospedaliero di Militello Val di Catania,

6) Presidio Ospedaliero S. Marta e S. Venera di Acireale,

7) Presidio Ospedaliero SS. Salvatore di Paternò,

8) Presidio Ospedaliero di Biancavilla,

9) Presidio Ospedaliero di Bronte.

## Competing interest

All authors have no conflicts of interest in this issue.
